# Personality of nonprofit organizations’ Instagram accounts and its relationship with their photos’ characteristics at content and pixel levels

**DOI:** 10.3389/fpsyg.2022.923305

**Published:** 2022-09-27

**Authors:** Yunhwan Kim

**Affiliations:** College of General Education, Kookmin University, Seoul, South Korea

**Keywords:** Instagram, nonprofit, organization, personality, Big-Five, photos

## Abstract

Nonprofit organizations (NPO) can utilize social networking sites (SNSs) for their activities. Like individual users, they can create SNS accounts, upload posts to show what they are doing, and communicate with other users. Thus, their accounts can be investigated from the same perspective of personality which has been one of the key lenses through which SNS posts of individual users was investigated. In the line of literature that analyzed the personality of non-human objects such as products, stores, brands, and websites, the present research analyzed the personality of NPOs’ Instagram accounts using an online AI service. Also, it investigated how their personality traits were related to the characteristics of the uploaded photos at content and pixel levels. The results of analysis of 223,446 photos on 177 Instagram accounts suggested that the personality of NPOs’ Instagram accounts can be summarized as being high in openness and agreeableness but low in extraversion and neuroticism. And it was found that openness and agreeableness were the personality traits that associated the most with the photo features. Also, the personality traits of NPOs’ Instagram accounts, except neuroticism, were predicted from the photo features with an acceptable level of accuracy. Implications of this research and suggestions for further research were presented.

## Introduction

Personality has been utilized to explain human behavior in various domain (Uher, 2008). In the context of social networking sites (SNSs), the personality of their users has been reported to be related to the characteristics of the posts they upload. The relationship has been mainly investigated in one of two ways. The personality traits of SNS users were measured and the according differences in their posts were examined (Kim and Kim, 2018), or the personality traits were predicted from the features of the posts they uploaded (Settanni et al., 2018). These investigations have contributed to the understanding of individual users’ online behaviors on SNSs.

Not only to individual users, this approach can be applied to organizational accounts of SNSs. Attempts have been made to understand the appearances and behaviors of various nonhuman objects, such as products (Dumitrescu, 2019), stores (Willems and Brengman, 2019), and websites (Jain and Yadav, 2019), from the perspective of personality. In this regard, the online behaviors of organizational SNS accounts can be examined from the perspective of personality. The accounts managed by organizations are similar to those run by individual users in their functionalities; they can upload posts, follow or become friends with other users, and like or comment on other users’ posts. Thus, the relationship between the personality of accounts and the characteristics of their posts can be investigated in the same manner both in organizational and individual accounts.

Meanwhile, social media photos have not received sufficient attention in terms of their relationships with uploaders’ personality. Social media data in text form have been mainly analyzed in the literature because they remain the most dominant form in social media posts. However, the importance of photo data has been rapidly increasing. One photo can express what a large amount of text can, and a growing number of users are using photo-centric SNSs such as Instagram as well as photo-uploading functions of existing SNSs such as Twitter and Facebook. In this vein, studies have analyzed photo data and explored how they are related to uploaders’ personalities (Segalin et al., 2017; Kanchana and Zoraida, 2020). However, their research samples were limited to individual users’ accounts, and it remains understudied how SNS photos in organizational accounts are related to the personality of the accounts. Notably, unlike texts, photos can be analyzed at two levels: the content (high) and pixel (low) levels. Photos can convey what is shown in the photos and how the pixels of the photos show it, and viewers can interpret the meaning of the photos in these two aspects. Thus, relationships between SNS photos and uploaders’ personality should be investigated at these two levels, but this has not been actively done especially concerning organizational accounts.

This study focuses on nonprofit organizations (NPOs), among many types of organizations, because they use social media as a main communication channel with the public and stakeholders (Wang and Yang, 2020). They usually do not have sufficient monetary and human resources for large-scale campaigns or mass media advertisements, so they use social media functions that enable them to communicate with many individuals quickly and cheaply. Additionally, their social media messages are usually consistent with their purpose of establishment and less concerned about external factors. This is less the case in other types of organizations such as political parties and businesses, because they must consider surrounding conditions such as political or market situations and actions of competing organizations.

The purpose of this study is to investigate how the personality of NPOs’ Instagram accounts is related to the characteristics of their photos at the content and pixel levels. To achieve this aim, we employ an online artificial intelligence (AI) service to measure the personality of organizational accounts by using the caption text attached to each Instagram photo. The personality measured using online data has been reported to have the same or higher level of accuracy (Youyou et al., 2015; Hinds and Joinson, 2019), and the AI service has been used in the literature for various purposes (Dutta et al., 2017; Balakrishnan et al., 2019; Kern et al., 2019; Yun et al., 2019; Whittingham et al., 2020; Kop et al., 2021). Additionally, photo features used in the literature to analyze social media photos are extracted at the content and pixel levels, and we examine how these features are associated with and whether the features can predict the accounts’ personalities.

The remainder of this paper is organized as follows. Studies on personality and social media posts, the personality of nonhuman objects, and NPOs’ social media posts are reviewed. Additionally, we describe how the research sample was selected, how the personality of NPOs was measured, and which photo features were used for analysis. The analysis results are presented, the implications and limitations of this research are discussed, and topics for further research are suggested.

## Literature review

### Analysis of nonprofit organizations’ social media posts

The characteristics of SNS posts that NPOs uploaded to their accounts has been a research subject for many years. Many studies have focused on the posts’ aim, which was mainly analyzed based on the information–community–action framework (Lovejoy and Saxton, 2012). This theoretical framework considers messages from organizations as doing one of the followings: spreading information about the organizations and their activities, conducting dialogic communication that can create relationships and build communities, or calling for particular actions such as donation, buying products, or attending events (Lovejoy and Saxton, 2012). Results in literature have suggested that NPOs use SNSs mainly for spreading information (Gao, 2016; Zhou and Pan, 2016; Qu, 2020). While some studies have shown the difference in SNS messages by culture (Waters and Lo, 2012) and emergency situations (Olson et al., 2019), a larger body of literature had reached a similar conclusion that one-way delivery of information was the dominant reason that NPOs use SNSs (Chung et al., 2020).

Other studies have provided insights into the more diverse aspects of SNS posts on the accounts of NPOs. For example, Campbell and Lambright (2020) focused on human service organizations, such as United Ways and Community foundations, and analyzed how SNS messages differed in these organizations from those of other types of NPOs. They found that human service organizations delivered more messages on taking action. Vedel et al. (2020) analyzed SNS posts of nonprofit healthcare organizations to show how they use SNSs to achieve their organizational goals. Their results suggest that the organizations use SNSs to replicate their existing websites, improve their engagement with the public, or employ novel functions of SNSs. Wang and Yang (2020) compared Twitter messages of nonprofit and for-profit organizations in terms of their dialogic relationship with their publics. They found that the emphasis of these two types of organizations differed: NPOs focused on the usefulness of information, while for-profit organizations focused on dialogic loops. Dineva et al. (2020) examined how NPOs managed the conflicts among consumers on their SNS pages. Their results from content analysis identified five strategies for managing conflicts, namely, non-engaging, censoring, bolstering, educating, and mobilizing.

Many studies, including those that we briefly reviewed, have focused on the characteristics of SNS posts uploaded by NPOs, and most of them have analyzed text data such as tweets. By contrast, the SNS data in photo form uploaded to NPOs’ accounts have not drawn much attention in the literature. Some studies have analyzed visual data on NPOs’ accounts (Guidry et al., 2017; Boscarino, 2022), but their analysis was mainly performed by human coders. This limited method prevented researchers from analyzing a large amount of data and investigating photo data at the pixel level, which is distinct from the content level, in which information is delivered and meanings are created. This research attempts to fill this gap and conduct a computational analysis of the Instagram photos uploaded to NPOs’ accounts.

### Personality of nonhuman objects

The attempt to understand the appearances and behaviors from the perspective of personality was expanded to various nonhuman objects. First, the personality of products was explored. Jordan (2002) pointed out that each product has a personality that differentiate it from other products, just as each individual can be differentiated by personality. This product personality influences designers’ selection concerning the product (Choi, 2017) and customers’ preference for the product (Prieto et al., 2020). Store personality has also been examined. For example, Hoa and Thao (2017) devised questionnaires for store personality, and showed that it comprised four dimensions, namely, sophistication, enthusiasm, economy, and reliability. Concerning online objects, the personality of websites was investigated in literature (Lal and Katole, 2021). Akrimi (2016) examined the personality of Internet service providers and showed that enthusiasm and genuineness were positively associated, while solidity and unpleasantness were negatively associated, with satisfaction with the websites. In their work regarding the personality of online tourism products stores, Rezaei et al. (2016) found that website personality had positive impacts on utilitarian web browsing, hedonic web browsing, and impulsive buying. Jain and Yadav (2019) reported that website personality positively influenced on visitors’ purchase intention.

However, organizational SNS accounts have not been actively investigated from the perspective of personality. According to the computers are social actors (CASA) paradigm (Nass et al., 1994; Nass and Moon, 2000), individuals perceive computers as having the same personality as humans because they apply human social rule when they interact with computers (Gambino et al., 2020; Lombard and Xu, 2021). In a similar vein, SNS users may perceive organizational accounts as being like a human, and this similarity enables this study to explore organizational SNS accounts from the perspective of personality. Based on this consideration, this study raises following research question:

RQ1. What are the characteristics of the personality of NPOs’ Instagram accounts?

### Personality of social media users and the characteristics of their posts

The Big Five personality model (Digman, 1990; McCrae and John, 1992) has been widely used to examine SNS users. It considers human personality as comprising five factors—openness, conscientiousness, extraversion, agreeableness, and neuroticism—and represents human personality by relative strengths of these factors.

The literature has examined the characteristics of SNS posts in terms of the difference according to the uploaders’ personality. For example, Pentina and Zhang (2017) found that Facebook users high in extraversion, agreeableness, and conscientiousness disclosed more positive emotions on their posts. Agarwal and Toshniwal (2020) reported that Twitter users high in extraversion and agreeableness revealed stronger leadership in their online behavior during natural hazards. Wang and Chen (2020) investigated relationships between the personality of CEOs and organizational performance: their results suggests that extraversion, agreeableness, and emotional stability (the inverse of neuroticism) were positively related to cost efficiency and profitability and that conscientiousness was negatively related to them. Miller (2020) examined how the Big Five personality traits were related to inappropriate posting by college students on Facebook and Twitter. Their results indicated that users with a higher level of conscientiousness posted less inappropriate content.

In this study, we attempt to apply this approach to the SNS accounts of NPOs. Although the posts on organizational SNS accounts have been actively analyzed (Yan et al., 2018; Liao et al., 2020; Wang and Yang, 2020; Liu et al., 2021), what has been relatively understudied is their relation to the accounts’ personality. The following research questions are raised:

RQ2. How are the personality traits of NPOs’ Instagram accounts associated with the characteristics of the photos uploaded to the accounts?

### Predicting personality from social media photos

The literature has utilized SNS data for predicting users’ psychological characteristics including personality. SNS data can be considered as digital traces of the users, and personality traits are reported to be linked more strongly with online behaviors than offline ones (Azucar et al., 2018). Thus, analyzing SNS data has much potential for an unobtrusive way of measuring personality (Settanni et al., 2018). Especially, predicting personality using photo data is known to generate better results than using text data (Ferwerda et al., 2016), and previous studies have adopted this approach.

A group of studies used a particular type of SNS photos for predicting personality: profile photos (Kanchana and Zoraida, 2020), selfies (Moreno-Armendariz et al., 2020), or the photos that users liked (Segalin et al., 2017). However, photos that users posted were reported to have more predictive power than those photos (Samani et al., 2018), and another group of studies used photos that users uploaded on their accounts. Ferwerda et al. (2016) predicted the Big Five personality traits of 113 Instagram users from 22,398 photos on their accounts. Similarly, Kim and Kim (2018) used 25,394 Instagram photos to predict the Big Five personality traits of 179 users. Samani et al. (2018) showed that the uploaders’ personality can be predicted more accurately when their photos on different platforms, Twitter and Flickr, are used together for prediction. Also, Mohammadiani and Sadeghi (2020) showed that the predictive performance can be improved using profile, posted, and liked images of Flickr users.

Based on these studies, this research predicts the personality of NPOs’ Instagram accounts. The photo features that were used to predict the personality of individual users’ accounts in the literature are extracted, and machine learning models are trained to show the features’ predictability on personality. The following research questions are pursued:

RQ3. How are the personality traits of NPOs’ Instagram accounts predicted from the characteristics of the photos uploaded to the accounts?

## Materials and methods

### Research sample

The list of NPOs was obtained from the Nonprofit Times’ Top 100 Nonprofits on the Web,^1^ The Global Journal’s Top 100 nongovernmental organizations (NGOs),^2^ and the NGOs affiliated with the United Nations.^3^ We visited the official webpage of each organization to obtain its Instagram account. If its Instagram account was not presented on the webpage, we searched for it on Google. Additionally, during this search, nonprofit organizations not on the list were found and added to the list. The organizations without Instagram accounts or whose uploaded posts were less than 30 were excluded from the research sample. As a result, 177 Instagram accounts of NPOs were selected as the research sample (Table 1), and all posts (photos and accompanying caption texts) were downloaded using Instagram scraper.^4^ For analysis, 223,446 posts were used.

**Table 1:** Instagram accounts of nonprofit organizations in the research sample.

Accounts
350org, aaasorg, aarp, aclu_nationwide, actionagainsthunger, actionaidusa, acumenorg, aei, aflatoun_international, als, alzassociation, amdiabetesassn, american_heart, americancancersociety, americanhumanist, americankidneyfund, americanredcross, americares, amnesty, amnh, amrefhealthafrica, antislaveryinternational, artinstitutechi, ashokachangemakers, aspca, atlanticcouncil, audubonsociety, barefootcollege, boyscoutsofamerica, bracworld, brothersbrotherfoundation, cambiahealth, careorg, catholicreliefservices, ccrjustice, centerconcern, cf_foundation, cfr_org, charitywater, childrenssurgeryinternational, christianbroadcastingnetwork, clevelandclinic, clintonfoundation, collegeboard, compassion, conservationorg, creativecommons, crisisgroup, csis, danafarber, deliveringgood, directrelief, doctorswithoutborders, dosomething, earthcharterinternational, environmental_defense_fund, experimentabroad, fareshareuk, feedingamerica, feedthechildrenorg, focusonthefamily, friends_intl, gatesfoundation, geneva.call, girlscouts, global_witness, globalfootprintnetwork, globalgiving, gramvikasodisha, habitatforhumanity, harlemchildrenszone, herorats, humanesociety, humanity_inclusion_us, humanrightscampaign, humanrightswatch, injazalarab, insidenatgeo, international_alert, internationalmedicalcorps, interpeace, ippnw_central, kennedycenter, kickstart_international, kiva.org, landesaglobal, leagueofwomenvoters, legacyintl, makeawishamerica, mapintl, mariestopes, mayoclinic, medicmobile, mentalhealthamerica, mercycorps, metmuseum, mfaboston, momaps1, montereybayaquarium, movember, mssociety, napfofficial, nationalwildlife, nature_org, ngowgg, npr, nrdc_org, nypl, oceana, one, oneacrefund, oneworldhealth, opensocietyfoundations, oregonzoo, oxfaminternational, panzifoundation, partnersinhealth, pathglobalhealth, pbs, peta, philamuseum, planinternational, plannedparenthood, prathameducation, prochoiceamerica, rainforestalliance, rare_org, refugees, reprievehq, rescueorg, roomtoread, rooseveltntwrk, rootcapital, rotaryinternational, rsfinternational, samaritanspurse, sandiegozoo, savethechildren, sfcg_, shrinershospitals, sierraclub, soroptimist, stepup4students, stjude, teachforamerica, ted, thebigissuefoundation, thecartercenter, thewcs, tostaninc, transparency_international, transparenthands, trevorproject, ul_ncfr, una.usa, unicef, unicefusa, unitednationshumanrights, unitedway, ushahidi, wainwrighthouseinc, water, waterforpeople, wemovement, wfpusa, wfuna, wfwpi, wgbh, wikileaks, wikipedia, witness_org, womensaction, world_wildlife, worldbank, worldvision, worldymca, worldywca

### Measuring personality of organizational accounts

The personality of each account was assessed using IBM Watson Personality Insights, which was selected due to of its ease of use.^5^ This service assesses the personality of the author of a given text (Hrazdil et al., 2021) based on the research about the relationship between language and personality (Fast and Funder, 2008; Hirsh and Peterson, 2009; Yarkoni, 2010), and it has been used in the literature to examine the personality of the author of SNS texts (Kern et al., 2019; Yun et al., 2019; Whittingham et al., 2020; Sakib et al., 2021; Gruda and Ojo, 2022). In this study, the caption texts of all Instagram photos uploaded to a given account were sent to the server *via* application programming interface (API), which returned the Big Five personality traits—openness, conscientiousness, extraversion, agreeableness, and neuroticism—of the account by providing a value between 0 and 1 for each trait.

### Instagram photo features

Photo features that have been used in the literature (Ferwerda et al., 2016; Ferwerda and Tkalcic, 2018; Kim and Kim, 2020; Kim and Lee, 2021) to analyze Instagram photos were used for analysis. The features were extracted at the content and pixel levels: the content-level features were content category and facial features, and the pixel-level features were pixel color features and visual features. Because the unit of analysis in this study is an account, the following features were extracted from each photo and averaged across all photos on a given account (except features in the content category, which are account-level metrics).

#### Content category

The category to which the content of a photo belongs was determined using Computer Vision API in Microsoft Azure Cognitive Services.^6^ For a photo sent to the server *via* API, its content was categorized into one of the 15 predetermined classes by the pretrained AI: the classes are *abstract*, *animal*, *building*, *dark*, *drink*, *food*, *indoor*, *others*, *outdoor*, *people*, *plant*, *object*, *sky*, *text*, and *transportation*. Next, the share of each class out of all photos on a given account was calculated. Thus, for example, if *people* of an account is 0.5, half of the photos uploaded to the account were of people. Additionally, the *Gini* coefficient was measured. Since it is a metric of the degree of concentration (Gini, 1912), it shows nondiversity in terms of the content category of the photos in an account.

#### Facial features

Features regarding human faces in a photo were extracted using Face API in Microsoft Azure Cognitive Services.^7^ First, the *number of faces* was the measure of how many faces appeared in a photo, *closeup* was the measure of the ratio of the size of the biggest face in a photo to the total size of the photo, and *face ratio* was the measure of the ratio of the sum of sizes of all faces in a photo to the total size of the photo. Next, *age* was the measure of the average age of the appearing faces, and *gender* was the measure of the number of female faces in a photo. Additionally, the emotions expressed on each appearing face were determined using Face API. The emotions include *anger*, *contempt*, *disgust*, *fear*, *happiness*, *sadness*, *surprise*, and *neutral*, and the sum of the eight emotions becomes 1 for a given face. The averages for each of the eight emotions on all faces in a photo were measured.

#### Pixel color features

Digital photos consist of pixels containing information on visual characteristics such as colors. It can be expressed by diverse color space models such as RGB (red, green, blue) and HSV (hue, saturation, value). Using this pixel-level information, we extracted the following features using the Python programming language and OpenCV library (codes are presented in Supplementary material).

First, the red, green, and blue in RGB were, respectively, averaged across all pixels in a photo, and their variances were also obtained. The same was performed for saturation and value (i.e., lightness) in HSV. As a result, the following features were measured: *red mean*, *red variance*, *green mean*, *green variance*, *blue mean*, *blue variance*, *saturation mean*, *saturation variance*, v*alue mean*, and *value variance*. Hue is a nominal feature unlike saturation and value. Thus, its total range (0 to 179 in OpenCV) was divided into intervals ([7, 23, 35, 90, 136, 169]) so that each interval corresponds to each key color (red, orange, yellow, green, blue, and violet), and the share of pixels whose hue falls into each color interval was calculated (Kim and Kim, 2018). As a result, the following features were measured: *red share*, *orange share*, *yellow share*, *green share*, *blue share*, and *violet share*. Also, the share of warm colors (red, orange, and yellow; *warm share*) and the share of cold colors (green, blue, and violet; *cold share*) were also measured. In addition, the number of peaks in a hue histogram (*hue peaks*) was measured: a hue histogram was generated, smoothed by kernel density estimation, and the number of local maximums was counted (Kim and Kim, 2018).

#### Visual features

The features concerning the visual attractiveness of a photo, suggested by San Pedro and Siersdorfer (2009) and used in the literature (Trattner et al., 2018; Messina et al., 2019; Yazdavar et al., 2020; Zhang et al., 2020), were extracted (see Table 2 for summary of visual features). First, *brightness*, which represents how bright a photo is, was measured by the average of luminance (Y values in the YUV color space) in the pixels of the photo. Next, *colorfulness*, which represents how colorful a photo is, was measured using the means and standard deviations of metrics composed of relative amounts of red, green, and blue values in the pixels. *Naturalness*, which represents how much a photo corresponds to the human perception of reality, was measured using the proportion of pixels whose saturation and luminance fall in a certain range. *Contrast*, which represents the relation of local luminance variations to the surrounding luminance, was measured by the standard deviation of luminance in pixels divided by the number of pixels. *RGB contrast* was also measured by extending contrast into the three-dimensional RGB color space. *Sharpness*, which represents a photo’s clarity and level of detail, was measured as a function of Laplacian of each pixel’s luminance, normalized by the local average luminance in the surroundings of each pixel. Two additional visual features concerning color were measured. *Color diversity*, which represents how diverse the colors used in a photo are, was measured by fractal dimension using the box-counting method (Feng et al., 1996); fractal dimension has been used as a metric of color diversity in the literature (Kim et al., 2014; Kim and Kim, 2019). *Color harmony*, which represents how harmonious the dominant colors in a photo are, was measured by the geometric formulations generated by the dominant colors on the color wheel; the highest and the second highest peaks in the smoothed hue histogram were identified as the top two dominant colors, and the internal angle made between the two colors on the color wheel is color harmony (Kim and Kim, 2019). Finally, *pleasure*, *arousal*, and *dominance* were measured by the affections from the PAD model using the formula^8^ in the literature (Valdez and Mehrabian, 1994).

**Table 2:** Summary of visual features.

Feature	Measures	Authors
Brightness	The average of luminance (Y values in the YUV color space)	San Pedro and Siersdorfer (2009)
Colorfulness	The means and standard deviations of metrics composed of relative amounts of red, green, and blue values in the pixels	
Naturalness	The proportion of pixels whose saturation and luminance fall in a certain range	
Contrast	The standard deviation of luminance in pixels divided by the number of pixels	
RGB contrast	Extending contrast into the three-dimensional RGB color space	
Sharpness	A function of Laplacian of each pixel’s luminance, normalized by the local average luminance in the surroundings of each pixel	
Color diversity	Fractal dimension using the box-counting method	Kim et al. (2014)
Color harmony	The geometric formulations generated by the dominant colors on the color wheel	Kim and Kim (2019)
Pleasure	The affections from the PAD model	Valdez and Mehrabian (1994)
arousal		
dominance		

## Results and discussions

### Mean personality traits of nonprofit organizations’ Instagram accounts (RQ1)

**Figure 1:**
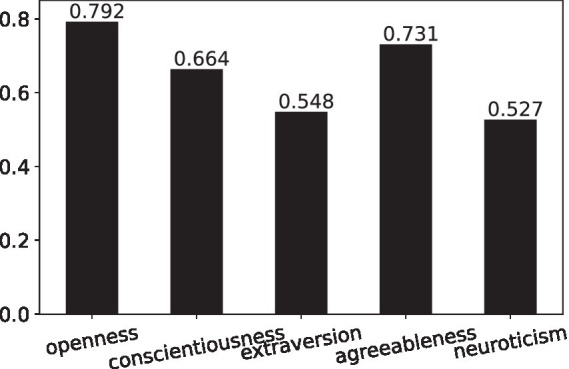
Mean personality traits of nonprofit organizations’ Instagram accounts in the research sample.

Figure 1 presents the mean personality traits of NPOs in the research sample for RQ1. It shows that openness and agreeableness were relatively high, while extraversion and neuroticism were relatively low. In other words, the personality of NPOs in the research sample can be summarized as being open and agreeable rather than extraverted and neurotic.

This pattern in Big Five personality traits of NPOs’ Instagram accounts was compared with the ones of other individuals and organizations in the literature which used IBM Watson Personality Insights. The personality of McDonald’s Twitter account was relatively high in extraversion and agreeableness while relatively low in openness and neuroticism, that of Harley-Davidson was relatively high in openness and conscientiousness while relatively low in neuroticism, and that of Tom’s Shoes was relatively high in extraversion and agreeableness while relatively low in neuroticism (Yun et al., 2019). Also, the personalities of Indian celebrities were measured from their tweets (Dutta et al., 2017): actors and sports figures were relatively high in extraversion, and politicians were relatively high in openness. Additionally, the personality of mass murderers measured from their writings was relatively high in openness while relatively low in extraversion and agreeableness (Kop et al., 2021).

These results are distinct from the pattern in the personality of NPOs’ Instagram accounts reported in this study. However, a similar pattern can be found in the personality of public health organizations’ Instagram accounts (Kim and Lee, 2021): this suggest that Instagram accounts of public health organizations and NPOs share the similar personality in common. This result may be explained by the characteristics of those organizations: public health organizations and NPOs usually aim to contribute to the public interest, and this might have made their online communication open and agreeable.

### Correlations between personality traits and photo features (RQ2)

Correlational analyses were conducted for RQ2 and the results are presented in Table 3. It shows that certain personality traits were mainly associated with certain kinds of photo features. Openness was the personality traits that associated the most with all kinds of photo features, and agreeableness was associated with many of content category, facial, and visual features. Additionally, neuroticism was associated mainly with facial features, and conscientiousness was associated mainly with pixel color features. As shown in the previous subsection, openness and agreeableness were the highest traits in the NPOs’ mean personality traits, and this can be the possible reason why they had significant correlations with many of photo features.

**Table 3:** Correlations between the personality traits of nonprofit organizations’ Instagram accounts and the features of their photos.

	Feature	Openness	Conscientiousness	Extraversion	Agreeableness	Neuroticism
Content category	Abstract	0.534^*^	−0.148^*^	0.228^*^	−0.298^*^	−0.021
	Animal	0.214^*^	−0.160^*^	−0.119	−0.174^*^	−0.115
	Building	0.375^*^	−0.020	0.185^*^	−0.163^*^	−0.035
	Dark	0.233^*^	−0.053	0.013	−0.280^*^	−0.134
	Drink	−0.030	0.171^*^	0.086	0.043	−0.002
	Food	0.044	−0.023	−0.111	−0.057	0.054
	Indoor	0.160^*^	−0.063	0.273^*^	−0.080	0.151^*^
	Others	0.309^*^	−0.197^*^	−0.206^*^	−0.299^*^	−0.131
	Outdoor	0.300^*^	−0.261^*^	−0.030	−0.434^*^	−0.210^*^
	People	−0.451^*^	−0.121	−0.141	0.328^*^	0.095
	Plant	0.306^*^	−0.205^*^	−0.004	−0.456^*^	−0.254^*^
	Object	0.540^*^	−0.198^*^	0.088	−0.259^*^	−0.002
	Sky	0.314^*^	−0.049	0.087	−0.260^*^	−0.110
	Text	−0.113	0.479^*^	0.189^*^	0.202^*^	0.103
	Transportation	−0.069	−0.047	−0.171^*^	0.131	0.025
	Gini	−0.538^*^	0.191^*^	−0.098	0.473^*^	0.107
Facial features	Number of faces	−0.495^*^	0.082	0.044	0.344^*^	0.077
	Face ratio	−0.321^*^	−0.045	−0.287^*^	0.374^*^	0.156^*^
	Closeup	−0.258^*^	−0.066	−0.321^*^	0.341^*^	0.153^*^
	Age	−0.313^*^	0.019	0.054	0.200^*^	0.117
	Gender	−0.428^*^	0.118	0.169^*^	0.342^*^	0.052
	Anger	0.006	−0.102	−0.076	−0.025	0.182^*^
	Contempt	−0.094	−0.133	−0.110	0.108	0.169^*^
	Disgust	−0.086	−0.226^*^	−0.130	0.025	0.105
	Fear	0.002	−0.098	−0.099	0.082	0.162
	Happiness	−0.588^*^	0.022	−0.073	0.454^*^	−0.041
	Sadness	−0.098	−0.203^*^	−0.287^*^	0.059	0.151^*^
	Surprise	−0.168^*^	0.011	0.016	0.186^*^	0.244^*^
	Neutral	−0.029	−0.146	−0.129	0.062	0.247^*^
Pixel color features	Red mean	−0.192^*^	0.181^*^	0.137	0.263^*^	−0.034
	Red variance	−0.381^*^	0.253^*^	0.126	0.279^*^	0.091
	Green mean	−0.161^*^	0.213^*^	0.130	0.147	−0.086
	Green variance	−0.312^*^	0.199^*^	0.140	0.240^*^	0.088
	Blue mean	−0.175^*^	0.338^*^	0.194^*^	0.213^*^	−0.004
	Blue variance	−0.284^*^	0.062	0.050	0.121	−0.036
	Saturation mean	−0.248^*^	−0.090	−0.094	−0.051	0.031
	Saturation variance	−0.405^*^	0.090	−0.085	0.134	0.022
	Value mean	−0.263^*^	0.265^*^	0.148^*^	0.244^*^	−0.036
	Value variance	−0.168^*^	0.028	0.072	0.101	0.078
	Red share	−0.205^*^	−0.090	−0.129	0.253^*^	0.066
	Orange share	0.073	−0.336^*^	−0.226^*^	0.030	−0.010
	Yellow share	0.107	−0.022	0.145	−0.125	−0.074
	Green share	−0.170^*^	−0.152^*^	−0.009	−0.147	−0.093
	Blue share	−0.094	0.130	−0.022	−0.057	0.060
	Violet share	–	–	–	–	–
	Warm share	0.041	−0.306^*^	−0.177^*^	0.051	−0.014
	Cold share	−0.185^*^	0.048	−0.027	−0.136	0.010
	Hue peaks	0.018	0.087	−0.012	0.007	−0.031
Visual features	Brightness	−0.181^*^	0.229^*^	0.147	0.200^*^	−0.064	
	Colorfulness	−0.410^*^	0.166^*^	0.022	0.205^*^	0.015	
	Naturalness	−0.214^*^	−0.131	−0.079	0.034	0.021	
	Contrast	−0.303^*^	0.104	0.059	0.194^*^	0.039	
	RGB contrast	−0.369^*^	0.146	0.069	0.225^*^	0.033	
	Sharpness	0.366^*^	−0.056	0.169^*^	−0.351^*^	−0.197^*^	
	Color diversity	−0.326^*^	−0.291^*^	−0.248^*^	−0.022	−0.084	
	Color harmony	−0.322^*^	−0.075	−0.182^*^	0.042	−0.059	
	Pleasure	−0.325^*^	0.243^*^	0.124	0.231^*^	−0.029	
	Arousal	−0.058^*^	−0.210^*^	−0.151^*^	−0.168^*^	0.043	
	Dominance	0.167^*^	−0.270^*^	−0.164^*^	−0.239^*^	0.042

* *p* < 0.05.

It was also found that openness and agreeableness showed correlations in opposite directions with content category features, facial features, and visual features. In other words, the features which were associated with openness positively (negatively) were associated with agreeableness negatively (positively). In terms of their meanings, openness and agreeableness are different but cannot be said to be opposite: the former is about curiosity and wide interests and the latter is being generous and sympathetic with others (McCrae and John, 1992). However, the findings suggest that these two traits were visually manifested in an opposite manner. Previous studies have reported that openness and agreeableness were associated in opposite directions with SNS photo features (Liu, 2016; Segalin et al., 2017; Matz et al., 2019; Kim and Lee, 2021), but that was not the case with SNS text features (Pentina and Zhang, 2017; Miller, 2020). Thus, it can be possibly presumed that the difference between the two traits is amplified to be opposite to each other when visually represented in SNS photos.

#### Openness and photo features

Table 3 shows that openness was significantly correlated with many of the content category features. Given that openness is related to curiosity and wide range of interests (McCrae and John, 1992), this diversity in content might be explained. The meaning of openness also corresponds with the negative association between openness and Gini, which indicates that the content of photos uploaded to the accounts of higher level of openness were more diverse. Notably, openness was negatively associated with the share of people photos. A possible explanation for this result may be the other aspect of openness which is related to artistic disposition (McCrae and John, 1992). Previous studies have reported that SNS users who are high in openness upload artistic photos (Kern et al., 2014; Liu, 2016), especially about abstract art (Feist and Brady, 2004), and these photos usually do not feature people (Matz et al., 2019). This may also hold for NPOs’ Instagram accounts. In a similar vein, less and smaller human faces appeared in the photos of accounts with higher openness (negative correlations with number of faces, race ratio, and closeup), and these results are consistent with previous studies (Liu, 2016; Matz et al., 2019).

Concerning pixel-level features, openness showed negative associations with means and standard deviations of RGB, saturation, and value. This was also the case with brightness, colorfulness, contrast, and color diversity. Those are the features whose high value might make the photos look fancy and splendid. These results suggest that the photos uploaded to the accounts with higher openness were generally darker, less strong in color, less bright, and less splendid. At first sight, it looks inconsistent with the artistic propensity in the definition of openness and the results of some previous studies (Fayn et al., 2015; Liu, 2016). However, other studies have suggested that the artistic propensity can be expressed in other ways including grayscale images (Guntuku et al., 2017), whose luminous and brightness were negatively associated with openness (Ferwerda et al., 2016), and this might be the possible explanation for the above results.

#### Conscientiousness and photo features

Conscientiousness is the propensity to leads one’s life in an efficient and well-organized manner, and this trait is related to being reliable and responsible (McCrae and John, 1992). The SNS photos of highly conscientious users were reported to be mainly of formal settings including office and classroom (Guntuku et al., 2017). These might be the reason why conscientiousness was positively associated with text but negatively with animal, outdoor, and plant in our results. Also, disgust and sadness were negatively associated with conscientiousness. This result may be explained by the above definition of conscientiousness, which can be linked to not expressing negative emotions in SNS photos (Liu, 2016; Bhatti et al., 2017).

The results in Table 3 also suggest that conscientiousness is related to the photos’ being strong in colors and luminous (positive correlations with RGB mean, value mean, colorfulness, and brightness). It is possibly because conscientious SNS users may prefer uploading posts in a normative and conventional manner, and they usually do not upload artistic or experimental photos as highly open users do (Guntuku et al., 2017). And these results correspond with the literature which has reported positive correlations of conscientiousness with RGB mean and value mean (Liu, 2016; Guntuku et al., 2017), colorfulness (Bhatti et al., 2017), and brightness (Liu, 2016).

#### Extraversion and photo features

Extraversion is the trait of being outgoing, talkative, and active and energetic in social interaction (McCrae and John, 1992), and the SNS posts of highly extrovert users have been reported to include social words and phrases like “party” and “cannot wait” (Kern et al., 2014). Thus, photos about people, social interaction (drink and food), and outdoor activities (outdoor, sky, plant, and transportation) can be expected to take large share in the accounts of high level of extraversion. On the contrary to this expectation, however, none of those content category showed significant correlations with extraversion. Rather, indoor, building, and text were positively associated with extraversion. This may be due to the characteristics of NPOs: they are organizations for social activities, so they may show their activities and opinions rather than leisure and pastime. Also, this might be why faces were small (negative associations with face ratio and closeup) and the color of photos were less diverse and less harmonious (negative associations with color diversity and color harmony) in the photos of highly extravert accounts.

#### Agreeableness and photo features

Agreeableness refers to the propensity of being generous, kind, and sympathetic with others (McCrae and John, 1992). This may be why photos of people-related content had large share in the highly agreeable accounts (positive association with people), as in previous studies (Guntuku et al., 2017). The negative associations with animal, outdoor, plant, and sky could also be attributed to this propensity because high agreeableness is known to prefer social interaction over nature and sustainable lifestyle (Saitov et al., 2021). And the photos on the highly agreeable accounts contained more and larger human faces (positive correlations with number of faces, face ratio, and closeup): these results are thought to be the reflections of the generous and sympathetic social interactions and correspond with the results of previous studies (Liu, 2016; Matz et al., 2019).

Concerning pixel-level characteristics, the photos of highly agreeable accounts were bright (positive associations with value mean and brightness), colorful (positive associations with colorfulness), and contrasting (positive associations with contrast). The generous, kind, and sympathetic propensity is consistent with these appealing characteristics of photos (Celli et al., 2014; Liu, 2016; Bhatti et al., 2017). Also, this propensity would make the affection of photos less aroused (negative association with arousal).

#### Neuroticism and photo features

Neuroticism, or emotional instability, is concerned with being anxious, worrying, touchy, and self-pitying (McCrae and John, 1992). This propensity can be the possible explanation for the positive associations with negative emotions such as anger, contempt, fear, sadness, and surprise, and neutral emotion. The associations between neuroticism and negative/neutral emotions on SNS posts have been reported in many previous studies (Golbeck et al., 2011; Schwartz et al., 2013; Kern et al., 2014; Liu, 2016). Also, the literature has found that neuroticism is closely linked to not presenting faces on SNS photos (Liu, 2016) and was associated negatively with the number and size of human faces on the photos (Celli et al., 2014; Matz et al., 2019). However, the results in this study contradict the literature: neuroticism was positively associated with face ratio and closeup. It suggests that the bigger (although not more) human faces appeared in the photos of highly neurotic accounts. This can be the characteristics of NPOs’ Instagram accounts because similar results are hardly found in the literature.

### Predicting personality traits using photo features (RQ3)

For RQ3, in addition to the correlational analyses, predictive models were built and analyzed to examine the predictability of the photo features on the personality traits of NPOs’ Instagram accounts. A random forest regressor with 10-fold cross-validation was trained for each personality trait, and a root mean square error (RMSE) was calculated to observe the predictability of each model (Table 4). These RMSEs were compared with those in the literature in which SNS user personalities were predicted from their photo features and the predictive power of the models were reported to be adequate. The RMSEs were 0.7–0.9 (Skowron, 2016), 0.66–0.78 (Ferwerda et al., 2016), or 0.561–0.737 (Kim and Kim, 2018), in which the personality traits were measured using 5-point Likert scale. For comparison, these RMSEs were divided by 4, the range of 5-point scale, to convert into the [0, 1] scale used in this study. As a result, the RMSEs of 0.5–0.7 in the 5-point scale were converted into 0.125–0.175 in the [0, 1] scale.

**Table 4:** Root mean square errors in 10-fold cross validation of random forest regression on personality traits.

	Openness	Conscientiousness	Extraversion	Agreeableness	Neuroticism
Content category	0.116	0.135	0.125	0.144	0.194
Facial features	0.112	0.139	0.123	0.146	0.199
Pixel color features	0.119	0.138	0.128	0.152	0.193
Visual features	0.119	0.142	0.131	0.150	0.192
All	0.111	0.134	0.121	0.140	0.187

We compared our RMSEs and those in the literature. Table 4 presents the results, suggesting that the predictive power of Instagram photo features on the Big Five personality traits, except neuroticism, of NPOs’ accounts was acceptable. This result is consistent with the literature suggesting that the personality of SNS users can be predicted from what they have written or uploaded to their accounts (Ferwerda et al., 2016; Azucar et al., 2018; Kim and Kim, 2018; Samani et al., 2018; Settanni et al., 2018). In addition, the result that neuroticism was not predicted from the features is also consistent with the literature which has suggested that neuroticism is the most difficult trait to predict (Ferwerda et al., 2016). These consistencies indicate that organizational SNS accounts can be investigated from the perspective of personality as has been performed for individual users’ accounts.

## Conclusion

The present study investigated the personality of NPOs’ Instagram accounts and according differences in their uploaded photos at content and pixel levels. It was found that the personality of NPOs was relatively high in openness and agreeableness but relatively low in extraversion and neuroticism. It was also found that openness and agreeableness were the personality traits which were associated the most the features of Instagram photos on NPOs’ accounts. And this study found that the personality traits of NPOs’ Instagram accounts, except neuroticism, can be predicted from the features of photos uploaded to the accounts.

### Implications of this study

The findings of this study imply that online behaviors of NPOs can be understood from the perspective of personality. We demonstrated that, similar to those of individual users, NPOs’ Instagram accounts differed in what they upload to their accounts on the basis of their personality. Thus, a SNS campaign by NPOs can be considered by the public to be a communication with an individual with a particular type of personality that is open and agreeable and not extraverted and neurotic. This can provide practical, expedient methods for designing SNS messages because messages that correspond more with organizations’ personalities are expected to have a stronger influence on their communication with the public. For example, NPOs would emphasize features that associated with openness and agreeableness but not those associated with extraversion and neuroticism, and this can increase the appeal of their Instagram photos.

### Limitations and suggestions for further research

The main limitation of this study is that its research sample comprised a limited number of NPOs. Further research which employs larger and diverse research sample can reveal the difference in the personality of NPOs by their activity area, countries, and culture. This study showed that the personality of NPOs’ Instagram accounts is similar with that of public health organizations’ Instagram accounts. Further research is expected to examine whether this similarity is general across organizational Instagram accounts or peculiar to those of particular types. Concerning the relationships between personality traits and photo features, future research may examine how the relationships differ by types of organizations. It is expected that more correlations and predictions are obtained and the relationships between personality traits and photo features in organizational SNS accounts are theorized.

In addition, it can be questioned that the personality of NPOs’ Instagram accounts can be attributed to the personality of account manager and/or of who are allowed to upload photos to the account. Future research can address this question by investigating whether the personality of account manager is directly represented on the accounts or other factors in organizations influence the accounts’ personality.

## Data availability statement

The original contributions presented in the study are included in the article/Supplementary material; further inquiries can be directed to the corresponding author.

## Author contributions

The author confirms being the sole contributor of this work and has approved it for publication.

## Conflict of interest

The author declares that the research was conducted in the absence of any commercial or financial relationships that could be construed as a potential conflict of interest.

## Publisher’s note

All claims expressed in this article are solely those of the authors and do not necessarily represent those of their affiliated organizations, or those of the publisher, the editors and the reviewers. Any product that may be evaluated in this article, or claim that may be made by its manufacturer, is not guaranteed or endorsed by the publisher.

## Footnotes

^1^ https://topnonprofits.com/lists/best-nonprofits-on-the-web/

^2^ http://theglobaljournal.net/top100ngos/

^3^ http://www.ngo.org/links/list.htm

^4^ https://github.com/arc298/instagram-scraper

^5^This service was deprecated as of 1 December 2021. Interested readers can consider other alternatives including: • https://github.com/senticnet/personality-detection (Majumder et al., 2017) • http://farm2.user.srcf.net/research/personality/recognizer.html (Mairesse et al., 2007) • Apply Magic sauce. https://applymagicsauce.com/

^6^ https://azure.microsoft.com/services/cognitive-services/computer-vision/

^7^ https://azure.microsoft.com/services/cognitive-services/face/

^8^Pleasure = 0.69 × Value + 0.22 × Saturation; Arousal = −0.31 × Value + 0.60 × Saturation; Dominance = −0.76 × Value +0.32 × Saturation.
