# Supplementary material for: Personality of nonprofit organizations’ Instagram accounts and its relationship with their photos’ characteristics at content and pixel levels
Source: Front Psychol. 2022 Sep 27;13:923305. doi: 10.3389/fpsyg.2022.923305 (PMC9551347; doi:10.3389/fpsyg.2022.923305)
Supplement: Supplementary file 1 [file Table_1.DOCX]

Supplementary Material

# Code for pixel color features

def bgr_metrics(image):

blue = image[:, :, 0]

blue_mean = blue.mean()

blue_var = blue.var()

green = image[:, :, 1]

green_mean = green.mean()

green_var = green.var()

red = image[:, :, 2]

red_mean = red.mean()

red_var = red.var()

return blue_mean, blue_var, green_mean, green_var, red_mean, red_var

def saturation_metrics(image_hsv):

saturation = image_hsv[:, :, 1]

saturation_mean = saturation.mean() # mean

saturation_var = saturation.var() # variance

return saturation_mean, saturation_var

def value_metrics(image_hsv):

value = image_hsv[:, :, 2]

value_mean = value.mean()

value_var = value.var()

return value_mean, value_var

def color_share(image_hsv):

hue = image_hsv[:, :, 0] # Hue ([0, 179])

hue = hue * 2 # now hue lies [0, 358]

bins = (1, 15, 45, 70, 180, 260, 339)

hist = np.histogram(hue, bins=bins)

red_share = (hist[0][0] + hist[0][-1]) / hue.size

orange_share = hist[0][1] / hue.size

yellow_share = hist[0][2] / hue.size

green_share = hist[0][3] / hue.size

blue_share = hist[0][4] / hue.size

violet_share = hist[0][5] / hue.size

warm_share = red_share + orange_share + yellow_share

cold_share = green_share + blue_share + violet_share

return red_share, orange_share, yellow_share, green_share, blue_share, violet_share, warm_share, cold_share

from scipy.stats.kde import gaussian_kde

import peakdetect

def num_hue_peaks(image_hsv):

hue = image_hsv[:, :, 0]

x_grid = np.linspace(0, 180, 181)

try:

pdf = gaussian_kde(hue.ravel())

pdf_evl = pdf.evaluate(x_grid)

maxtab, mintab = peakdetect.peakdet(pdf_evl, 0.005)

return len(maxtab)

except Exception as e:

return 0
